# JiaWeiDangGui Decoction Ameliorates Proteinuria and Kidney Injury in Adriamycin-Induced Rat by Blockade of TGF-*β*/Smad Signaling

**DOI:** 10.1155/2016/5031890

**Published:** 2016-06-14

**Authors:** Ming-gang Wei, Wei-ming He, Xun Lu, Li Ni, Yan-yu Yang, Lin Chen, Pei-hua Xiong, Wei Sun

**Affiliations:** ^1^The First Affiliated Hospital of Soochow University, Suzhou, Jiangsu 215006, China; ^2^Jiangsu Province Hospital of Traditional Chinese Medicine, Nanjing, Jiangsu 210029, China

## Abstract

JiaWeiDangGui (JWDG) decoction has anti-inflammatory and antifibrotic effects, which is used widely for the treatment of various kidney diseases. In previous studies, we have found that JWDG decoction can reduce the quantity of proteinuria, but the mechanism was unknown. Here, we studied the protective effect of JWDG decoction in adriamycin-induced nephropathy on rat. JWDG decoction, at 10 mL/kg/d, 20 mL/kg/d, and 40 mL/kg/d, was orally administered daily for 12 weeks. Therapeutic effects and mechanisms were further examined. The kidney function related biochemical indexes were measured by automatic biochemistry analyzer. The pathomorphological changes were observed using light and transmission electron microcopies. The proteins expressions of podocin, nephrin, collagen IV, and fibronectin (FN) were examined by immunohistochemical staining, and key proteins involved in TGF-*β*/Smad signaling were evaluated by RT-PCR and western blotting. Compared with vehicle-treated controls, JWDG decoction decreased the quantity of proteinuria; reduced glomerulosclerotic lesions induced by ADR; and preserved the expression of podocin and nephrin. JWDG decoction also inhibited the expression of the collagen IV, FN, and fibrogenic TGF-*β*. Further studies revealed that inhibition of renal fibrosis was associated with the blockade of TGF-*β*/Smad signaling and downregulation of snail expression dose dependently. JWDG decoction prevents proteinuria production, podocyte dysfunction, and kidney injury in adriamycin nephropathy by inhibiting TGF-*β*/Smad signaling.

## 1. Introduction

Chronic kidney diseases (CKD) are emerging as a public health problem worldwide [1]. CKD is characterized by a progressive loss of renal function, associated with reduction of glomerular filtration rate and massive accumulation of proteinuria. Adriamycin-induced nephropathy is a classic rat model of chronic kidney disease which is considered to be an experimental analogue of human minimal lesion nephrotic syndrome, leading to chronic proteinuria and renal failure [2–4]. Nephrotic syndrome shows symptoms of massive proteinuria, hypoalbuminemia, and edema. Glomerular filtration barrier damage plays a critical role in proteinuria in CKD, which contains fenestrated endothelium, glomerular basement membrane (GBM), and podocytes [5, 6]. It has been shown that podocytes play an important role in maintaining the integrity of the glomerular filtration barrier. The podocyte injury is one of the major causes leading to defective glomerular filtration, which results in proteinuria. In recent years, several slit diaphragm related proteins such as nephrin, podocin, and CD2-associated proteins (CD2AP) have been identified. Disruptions in nephrin and podocin have been identified as the leading cause of several inherited and acquired forms of proteinuria [7–9]. It has been well documented that proteinuria not only is a marker for the progression of CKD but also acts as a pathogenic mediator that incites renal inflammation and promotes tubular injury and interstitial fibrosis [10–12]. TGF-*β* has been long considered as a key mediator in renal fibrosis [13–16]. The expression of TGF-*β* is universally upregulated in a wide variety of CKD in experimental and clinical settings. TGF-*β* initiates its fibrosis activity through the Smad protein signaling pathways, which play crucial roles in renal fibrosis [17, 18]. Therefore, the restoration of podocyte and the inhibition of TGF-*β*/Smad signaling will be potential strategies for reversing progression of CKD.

In China, traditional Chinese medicine (TCM) is widely used for the treatment of various kinds of kidney diseases [19] and has become a promising source of new therapeutic agents for CKD. JWDG decoction (also called Qiguiyishen decoction) is derived from Astragali Radix (Huangqi; AR) (roots of* Astragalus membranaceus* (Fisch.) Bunge), Angelicae Sinensis Radix (Danggui; ASR) (root of* Angelica sinensis* (Oliv.) Diels), Ligusticum chuanxiong (Chuanxiong; LC) (root of* Ligusticum chuanxiong* S. H. Qiu, Y. Q. Zeng, K. Y. Pan, Y. C. Tang & J. M. Xu), and Radix Achyranthis Bidentatae (Niuxi; RAB) (root of* Achyranthes bidentata* Blume), which are the most widely used herbs in TCM for treating kidney diseases [20]. Astragali Radix can reduce proteinuria and decrease the levels of TGF-*β*1 and fibroblast activation when combined with Angelicae Sinensis Radix [21–23]. Radix Achyranthis Bidentatae can promote blood circulation, which has been widely used to treat kidney diseases [24]. Ligusticum chuanxiong is an effective medical plant with antioxidation, antifibrosis, and anti-inflammation effects, which has been extensively applied to treat various diseases with other Chinese herbal medicines for many years [25].

We reported previously that treatment with JWDG decoction significantly reduced the accumulation of extracellular matrix in ADR-induced nephrotic syndrome in rats. In the present study, we sought to investigate the role of JWDG decoction in reducing proteinuria, protecting podocytes, and restoring the kidney function in an ADR-induced rat model.

## 2. Materials and Methods

### 2.1. Preparation of JWDG Decoction

The JWDG decoction was prepared as described previously [20], and herbs were purchased from Suzhou Tianling Chinese Herbal Medicine Co. Ltd (Suzhou, China). Briefly, JWDG extract was prepared using the following 4 herbs: Huangqi (Radix Astragali Mongolici) 600 g, Danggui (Radix Angelicae Sinensis) 200 g, Niuxi (Radix Achyranthis Bidentatae) 200 g, and Chuanxiong (Rhizoma Chuanxiong) 200 g. Herbs were boiled for 30 min after soaking in 2000 mL distilled water for 30 min. The decoction was further filtered and condensed to 1000 mL and stored at 4°C before use. For administration of JWDG decoction, the extract was suspended in 0.9% NaCl.

### 2.2. Expermiental Design

Experiments were performed in 60 adult male Sprague-Dawley rats (Certificate No. SCXK (shang hai) 2012-0002), weighing between 180 g and 220 g sourced from Shanghai Laboratory Animal Center. All rats were housed under standard temperature (23°C ± 1°C), relative humidity (55%  ± 10%), and 12 h light/12 h dark cycle with access to food and water ad libitum. Except for 10 rats of the normal group, all the other rats were given 6.5 mg/kg Adriamycin (dissolved in saline) via a single intravenous injection from the tail respectively [2]. Rats in the normal group were injected with saline only. After it was established that proteinuria was detected, the model rats were randomly assigned to four groups: model, and JWDG treatments with dose of 10, 20 and 40 mL/kg/d, respectively. The dosages applied was based on clinical and our preliminary experiments. Rats in normal and model groups were orally administrated with 10 mL/kg/d of saline. The experimental period was twelve weeks. During the course of the experiment, rats were housed in metabolic cages at different time points for the collection of 24-hour urine samples. Proteinuria was expressed as total urinary protein over creatinine. Rats were anesthetized before sacrifice and blood was collected from the inferior vena cava. Kidneys were harvested and processed for immunohistologic evaluation. Serum albumin, urinary creatinine and total protein were analyzed by automatic biochemical analyzer. The protocol in this study was approved by the Ethics Committee of Soochow University and performed in accordance with the NIH Guiding Principles for the Care and Use of Laboratory Animals (No. 2012-0045).

### 2.3. Histologic Examination

Transversal slices of kidneys were fixed in 4% buffered formaldehyde at 4°C for 24 hours and embedded in paraffin. Three-micrometer sections were cut and stained with hematoxylin-eosin (H&E), periodic acid Schiff (PAS), and Masson's trichrome, observed under the light microscopy. Glomerulosclerosis was graded on a scale of 0 to 4, with 0 indicating normal, 1 indicating 1% to 10% of glomeruli with sclerotic lesions, 2 indicating 11% to 25% of glomeruli with sclerotic lesions, 3 indicating 26% to 50%, and 4 indicating 50% of sclerotic glomeruli.

Renal tissue samples (1 mm^3^) for electron microscopic assessment were fixed in 2.5% glutaraldehyde. After washing in PB and postfixing in 1% osmium tetroxide, the fixed material was dehydrated in graded ethanol solutions and embedded in epoxy resin. Ultrathin sections were stained with uranyl acetate and lead citrate and then examined under a transmission electron microscope (JEM-1011, JEOL, Tokyo).

### 2.4. Immunohistochemistry

Immunohistochemical staining was performed using a routine protocol. Formalin-fixed, paraffin-embedded sections (3 *μ*m) were deparaffinized and rehydrated. Following antibodies were applied overnight at 4°C, including anti-fibronectin (ab23751, Abcam, Cambridge, UK), anti-collagen IV (ab6586, Abcam, Cambridge, UK), anti-podocin (ab93650, Abcam, Cambridge, UK), and anti-nephrin (ab183099, Abcam, Cambridge, UK). Anti-rabbit (goat polyclonal) antibodies were applied subsequently for 30 min. Slides were visualized using an Olympus light microscope, and quantification of intensity for each condition was performed using ImageJ software.

### 2.5. RNA Extraction and Quantitative RT-PCR Analysis

Renal cortex was collected by carefully removing the renal pelvis and medullar tissues and rapidly stored in −80°C. Total RNA of kidney tissues was extracted using Trizol reagent (Invitrogen, Life Technologies, Carlsbad, CA, USA) following the manufacturer's instructions. The synthesis of cDNA was performed using reverse transcriptase. Primers used for gene analysis were TGF-*β*1 forward 5′-ACGTCAGACATTCGGGAAGCAGTG-3′ and reverse 5′-GCAAGGACCTTGCTGTACTGTGTG-3′. The transcript levels of the target gene were normalized with *β*-actin gene.

### 2.6. Western Blot Analysis

Renal cortical tissues were lysed with radioimmunoprecipitation assay (RIPA) buffer, and proteins were extracted for western blot analysis as described previously [26]. After proteins were transferred to a polyvinylidene difluoride (PVDF) membrane, it was further blocked with 5% (w/v) non-fat milk in Tris buffered saline (TBST) for 1 h at 37°C. Blots were probed with diluted primary antibody, including anti-TGF-*β*1 (ab25121, Abcam, Cambridge, UK), anti-pSmad2/3 (ab63399, Abcam, Cambridge, UK), anti-Smad2/3 (ab63672, Abcam, Cambridge, UK), anti-snail (ab180714, Abcam, Cambridge, UK), and *β*-actin (Santa Cruz Biotechnology, Inc.). After hybridization, the membrane was washed and hybridized with 1 : 5000 (v/v) dilutions of goat anti-rabbit IgG, horseradish peroxidase-conjugated secondary antibody (Santa Cruz Biotechnology, Inc.). The signal was generated by adding enhanced chemiluminescent reagent. *β*-actin was used as an internal control. Ratio for the protein examined was normalized against *β*-actin and expressed as mean ± SD.

### 2.7. Statistical Analysis

Data were shown as mean ± SD. The statistical difference between groups was determined by the paired Student's *t*-test. A *P* value less than 0.05 was considered significant.

## 3. Results

### 3.1. JWDG Ameliorates Proteinuria and Renal Dysfunction in Adriamycin Nephropathy

**Figure 1:**
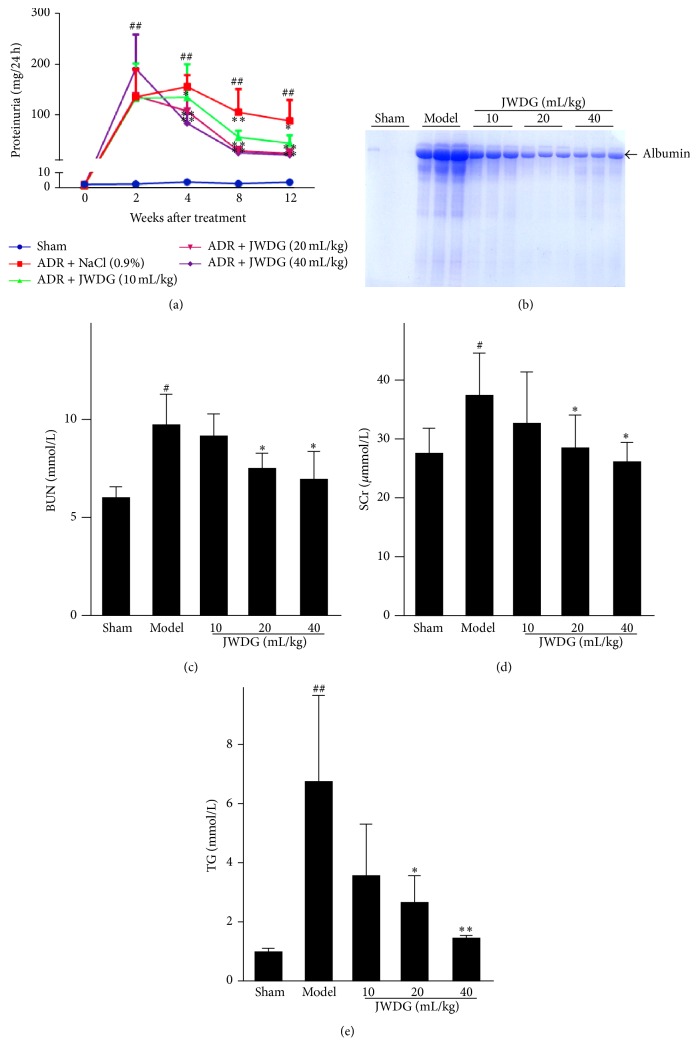
JWDG ameliorates proteinuria and renal dysfunction in adriamycin nephropathy. (a) Kinetics of 24 h urinary protein excretion in different groups. (b) SDS-PAGE analysis shows the composition of urinary proteins in different groups of rats at 12 weeks after ADR injection. The levels of BUN (c), Scr (d), and TG (e) in serum in different groups. Data represent groups of 8 rats and are expressed as mean ± SD. ^#^ *P* < 0.05, ^##^ *P* < 0.01 versus sham group. ^*∗*^ *P* < 0.05, ^*∗∗*^ *P* < 0.01 versus ADR group.

We investigated effects of JWDG in the ADR nephropathy, a model characterized by initial podocyte injury and albuminuria and subsequent renal inflammation and fibrosis. As shown in Figures 1(a) and 1(b), animals injected with ADR (model group) developed severe proteinuria compared with controls. The application of JWDG significantly prevented the production of proteinuria in a dose-dependent manner, especially with moderate (20 mL/kg/d) and high dosages (40 mL/kg/d). ADR-induced nephrotic syndrome is accompanied with renal dysfunction. Plasma triglyceride (TG), serum creatinine (SCr), and blood urea nitrogen (BUN), known as three important indicator indexes in renal function, were examined in this study. The result showed that ADR increased the levels of TG, SCr, and BUN significantly (*P* < 0.05). Interestingly, the amount of TG, SCr, and BUN was decreased after JWDG treatment for 12 weeks and dramatic inhibition was observed on rats applied with moderate (20 mL/kg/d) and high dosages (40 mL/kg/d) of JWDG, suggesting that JWDG could enhance renal function by reducing the synthesis or increasing the excretion of TG, SCr, and BUN in ADR-induced nephritic syndrome rats (Figures 1(c)–1(e)).

### 3.2. JWDG Attenuates Glomerular Lesions and Podocyte Injury In Vivo

**Figure 2:**
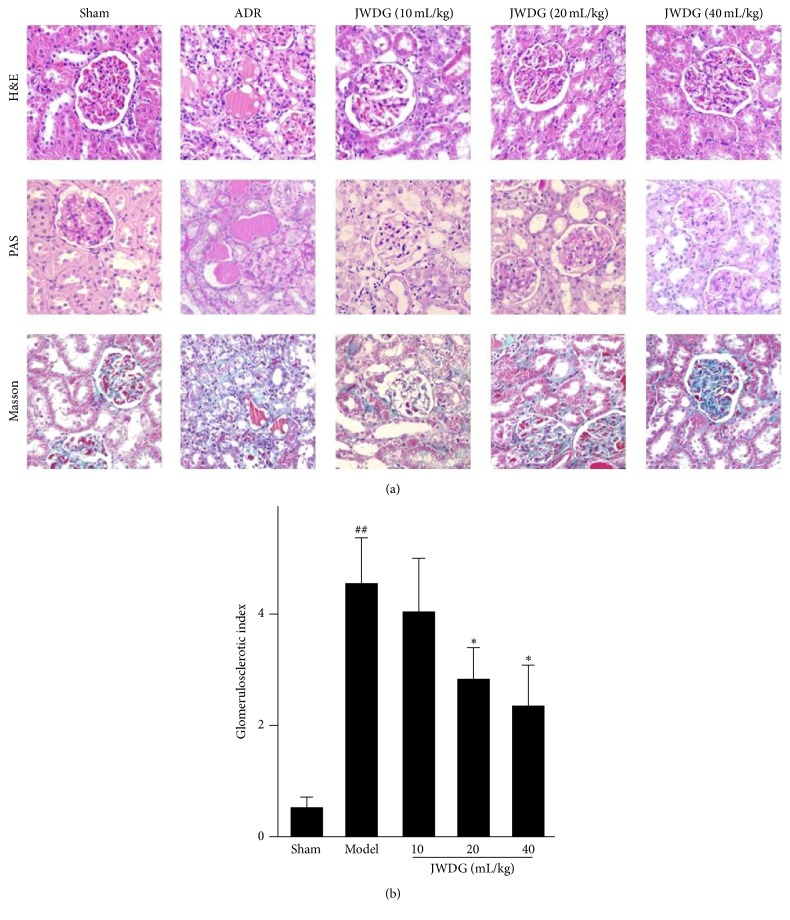
JWDG attenuates glomerular lesions in vivo. (a) Representative micrographs demonstrate kidney injury at 12 weeks after ADR injection in different groups. Kidney sections were subjected to H&E, periodic acid Schiff (PAS), and Masson's trichome-stained sections. (b) The glomerulosclerotic index. Data represent groups of 8 rats and are expressed as mean ± SD. ^##^ *P* < 0.01 versus sham group. ^*∗*^ *P* < 0.05 versus ADR group.

**Figure 3:**
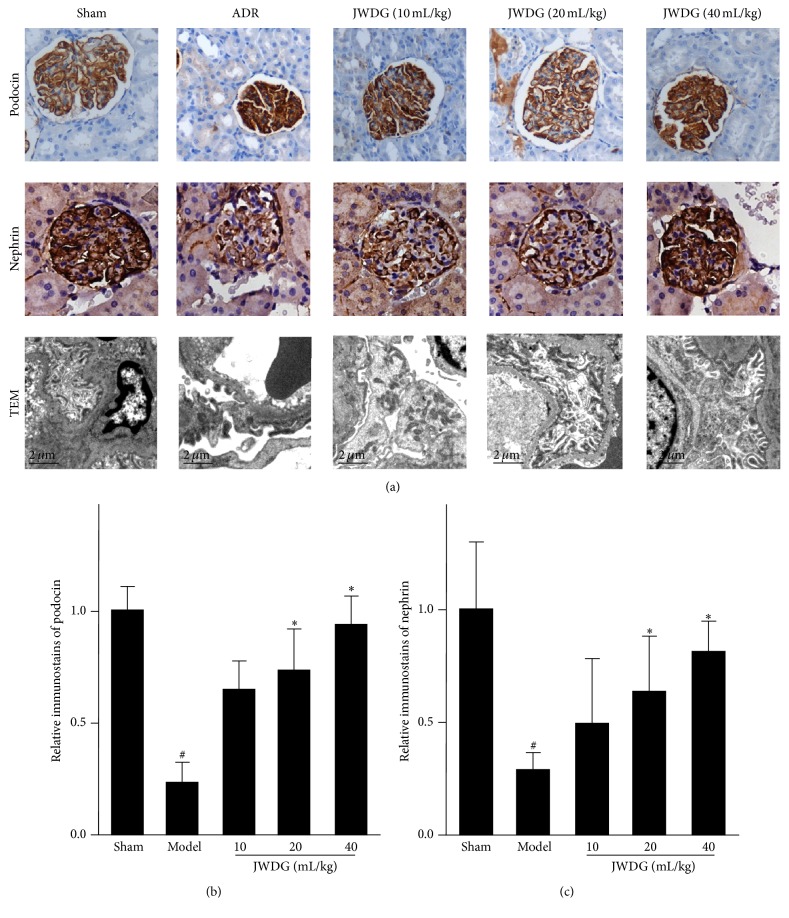
JWDG attenuates podocyte injury in vivo. (a) Immunohistochemistry of podocin and nephrin expression in different groups and electron microscopy of podocytes. The quantitative analysis of podocin (b) and nephrin (c). Data represent groups of 8 rats and are expressed as mean ± SD. ^#^ *P* < 0.05 versus sham group. ^*∗*^ *P* < 0.05 versus ADR group.

H&E staining results showed that kidney tubular lumen expansion and epithelial cell swelling were detected in the model group and reduced after treatments with JWDG for 12 weeks (Figure 2(a)). Kidney histology by PAS and Masson trichrome staining revealed clearly visible nephropathy at 12 weeks after ADR injection, associated with the development of glomerulosclerosis, thickening of glomerular basement membrane, tubular damage, and interstitial fibrosis. After treatments with JWDG, histological damage was ameliorated in a dose-dependent manner, especially applied with moderate (20 mL/kg/d) and high dosages (40 mL/kg/d) of JWDG (Figure 2(a)). A semiquantitative glomerulosclerotic index of kidney sections confirmed the histological data. The ADR group showed the highest score, and JWDG treatments led to a marked reduction in the index (*P* < 0.05) (Figure 2(b)). Because podocyte injury is an early and predominant pathologic feature in this model, we next investigated the protective effect of JWDG on podocyte in vivo. As shown in Figure 3, comparing with normal control, two proteins, nephrin and podocin, were substantially downregulated in the kidney at 12 weeks after ADR injection but upregulated after JWDG treatment, indicating an effective preservation of podocyte integrity. We also investigated the ultrastructural changes in podocytes by electron microscopy. The result showed that, in the model group, the foot process was extensive effacement and the width of foot process was much bigger than that from the normal group. Fortunately, JWDG could suppress the foot process effacement and reduce the width of foot process (Figure 3).

### 3.3. JWDG Reduces Renal Fibrotic Lesions after Adriamycin Injury

**Figure 4:**
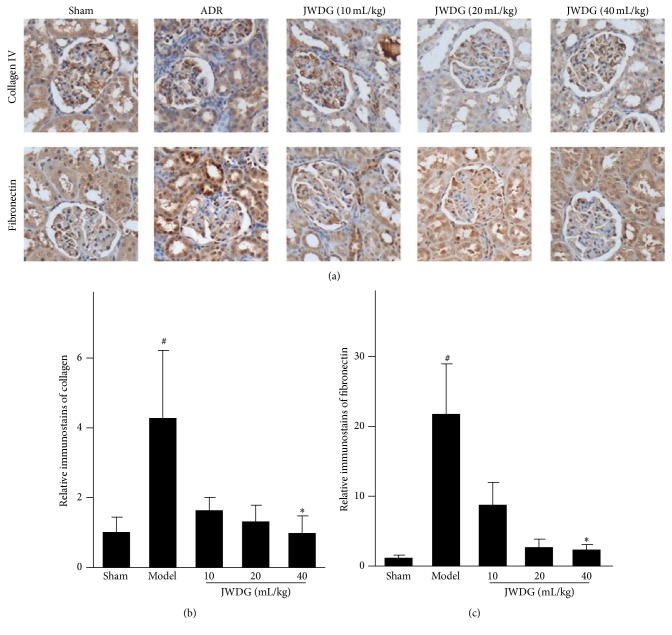
JWDG reduces renal fibrotic lesions. (a) Immunohistochemistry of collagen IV and fibronectin expression in different groups. The quantitative analysis of collagen IV (b) and fibronectin (c). Data represent groups of 8 rats and are expressed as mean ± SD. ^#^ *P* < 0.05 versus sham group. ^*∗*^ *P* < 0.05 versus ADR group.

**Figure 5:**
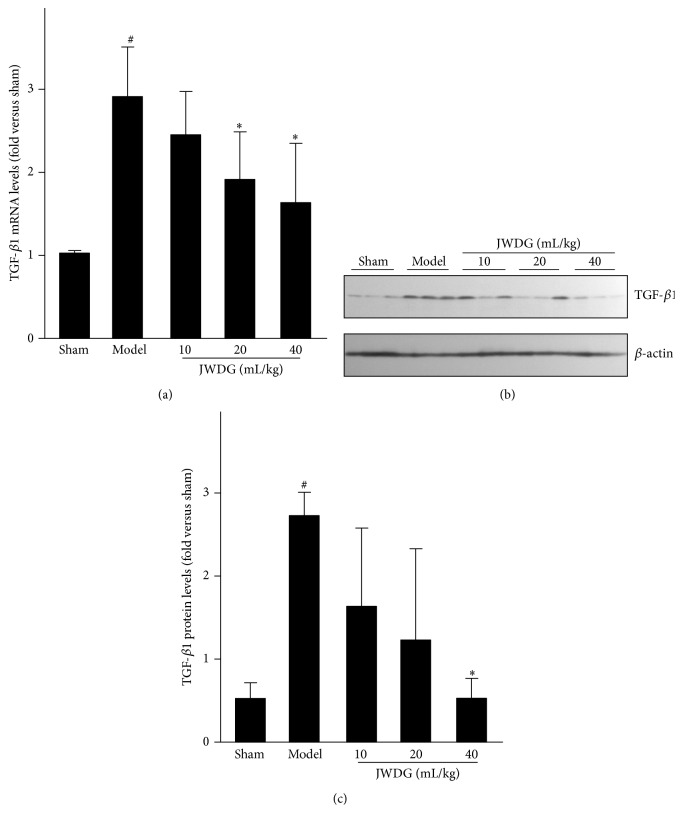
JWDG reduces TGF-*β*1 expression in mRNA and protein level. Real-time PCR (a), western blots (b) and quantitative analysis of TGF-*β*1 expression (c). Data represent groups of 8 rats and are expressed as mean ± SD. ^#^ *P* < 0.05 versus sham group. ^*∗*^ *P* < 0.05 versus ADR group.

Because ADR injury inevitably leads to renal fibrotic lesions, we next examined the effects of JWDG on renal fibrosis in this model. We examined the expression of renal collagen IV and fibronectin in different groups by immunohistochemical staining. As shown in Figure 4(a), renal collagen IV and fibronectin protein levels were dramatically increased after ADR injury, suggesting myofibroblast was activated in this model. However, the induction of these proteins was significantly abolished by JWDG. These results are consistent with the deposition of altered renal collagen revealed by Masson trichrome staining (Figure 2(a)). We further investigated the expression of TGF-*β*1, which was involved in the pathogenesis of CKD. As shown in Figure 5(a), RT-PCR analysis demonstrated that TGF-*β*1 mRNA level was increased in the kidney at 12 weeks after ADR injection; however, JWDG significantly inhibited the expression of TGF-*β*1. Similarly, the production of renal TGF-*β*1 protein was also induced after ADR injury but depressed by JWDG (Figures 5(b) and 5(c)).

### 3.4. JWDG Blocks TGF-*β*1/Smad Activation and Suppresses Its Downstream Snail Expression

**Figure 6:**
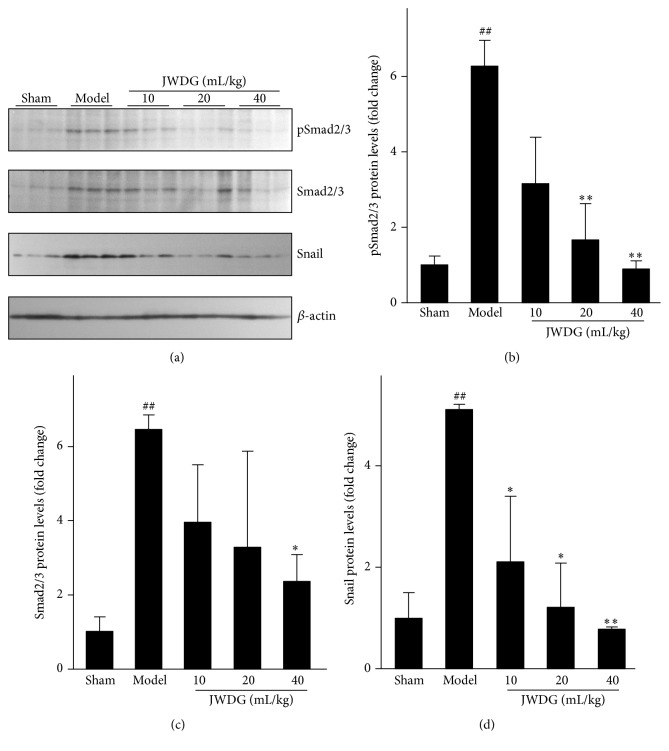
JWDG blocks TGF-*β*1/Smad activation and suppresses its downstream snail expression. Western blot (a) and quantitative analysis of renal pSmad2/3 (b), Smad2/3 (c), and snail (d) expression. Data represent groups of 8 rats and are expressed as mean ± SD. ^##^ *P* < 0.01 versus sham group. ^*∗*^ *P* < 0.05, ^*∗∗*^ *P* < 0.01 versus ADR group.

TGF-*β*1 is a potent fibrotic factor that is responsible for the synthesis of ECM in the kidney. Above results showed that the expression of TGF-*β*1 was significantly higher from rats in ADR group than those from sham group, and significant reduction of TGF-*β*1 was observed in JWDG group (40 mL/kg/d). We further investigated the effect of JWDG on protein expressions about active signaling molecules in TGF-*β*1/Smad pathway. Western blotting analysis showed that phosphorylated Smad2/3 expression was remarkably reduced after JWDG treatment in kidneys of rats (Figure 6). We also investigated the level of snail, an important downstream mediator of TGF-*β*1/Smad signaling pathway (Figure 6). Our results showed that treatment of JWDG in ADR rats could attenuate TGF-*β*1-induced upregulation of snail expression.

## 4. Discussion

CKD is a pathophysiologic process characterized by reduced glomerular filtration and persistent massive proteinuria. CKD has become an important research field, with its increasing morbidity and mortality [27]. JWGD decoction is a TCM, widely used for the treatment of various renal diseases. In this study, we examined the renal protective effects of JWDG in ADR nephropathy, a model characterized by initial podocyte injury, proteinuria, and late-onset renal inflammation and fibrosis.

Given the inherent nature of ADR nephropathy, we primarily studied the function of JWDG on ameliorating the proteinuria and renal and podocyte dysfunction. Proteinuria is a hallmark of kidney diseases and a risk factor causing the loss of kidney function in patients with CKD [28]. We found that an injection of 6.5 mg/kg^−1^ of ADR in the rat significantly induced proteinuria after 14 days; however, the production of proteinuria was dramatically reduced by JWDG dose-dependently. The pathological change of renal was observed by H&E staining, showing that JWDG can alleviate the kidney tubular lumen expansion and epithelial cell swelling caused by ADR treatment. The production of TG, SCr, and BUN was investigated as they are three important components reflecting renal function. In this study, notable increase of TG, SCr, and BUN was found in rats belonging to model group, indicating that renal filtrating function was impaired by ADR. Renal dysfunction reduces the capability of filtering them, and their level then rises [29]. Our results also showed that JWDG could decrease the levels of TG, SCr, and BUN in serum, indicating that JWDG could enhance renal function by reducing the synthesis or increasing the excretion of them in ADR-induced NS rats. Podocyte function of maintaining the filtration barrier of glomerulus depends on nephrin, podocin, and CD2-associated proteins, which are involved in maintaining the structural integrity of the slit diaphragms. The electron microscopy study demonstrated that JWDG not only prevented the loss of podocyte-specific nephrin and podocin, but also protected the integrity of podocytes, indicating that beneficial effects of JWDG were likely mediated by its ability to preserve podocyte integrity. The renoprotective effect of JWDG may be primarily attributable to its prevention of podocyte injury.

As we know, inflammation is an important component of the pathophysiology of ADR nephropathy [30, 31]. Previous studies have showed that key components of JWDG decoction, such as Astragaloside IV [32], ferulic acid [33, 34], oleanolic acid [35], and ligustrazine [36], have anti-inflammation effects in various kidney diseases. Then we investigated the anti-inflammation effects of JWDG on ADR model rats. Histological assessment of kidneys from ADR-treated rats showed that tubulointerstitial inflammation with infiltration of T and B lymphocytes and macrophages. In addition, most of ADR model rats were developed to have the typical glomerulosclerotic injury, such as the severe ECM accumulation and the significant glomerular cells proliferation. All features could be restored by application of medium to high dosage of JWDG. We further investigated the expression level of collagen IV and fibronectin, in the cortex of kidneys detected by semiquantitative immunohistochemical analysis. The results showed that the expressions of collagen IV and fibronectin in the cortex of the kidney in the JWDG treatment group were decreased compared with the untreated group, especially in the high dosages (40 mL/kg/d) group. Inflammation promotes progressive renal fibrosis, and TGF-*β*1 has been regarded as a key mediator in the progression of fibrosis. TGF-*β*1 was upregulated in ADR model, associated with increased synthesis of ECM. The downstream signaling effects of TGF-*β*1 were mediated by Smad [18]. Our results showed that the expression of TGF-*β*1 was upregulated in ADR group consistent with previous reports [27] but inhibited by JWDG. Therefore, we investigated the phosphorylation of Smad2 and Smad3. With the decrease of TGF-*β*1 expression, JWDG treatment significantly reduced the phosphorylation of Smad2/3. The results indicated that the antifibrogenic effect of JWDG was associated with the downregulation of TGF-*β*1 signaling pathway. Snail is the one well characterized and mostly relevant gene in many TGF-*β*/Smad downstream target genes, which played the important role in proteinuria and renal fibrosis observed in ADR nephropathy. Indeed, the expression of renal snail was significantly induced after ADR injection but suppressed by JWDG substantially.

## 5. Conclusion

In conclusion, our results revealed that JWDG decoction known as a Chinese traditional plant medicine could dose-dependently ameliorate proteinuria, podocyte injury, and renal fibrosis in ADR nephropathy, inhibiting the activation TGF-*β*/Smad pathway and snail expression. The therapeutic efficacy of JWDG decoction in ADR nephropathy is impressive, which might be a promising alternative therapy for nephrotic syndrome.
